# Tapered, Double-Lead Threads Single Implants Placed in Fresh Extraction Sockets and Healed Sites of the Posterior Jaws: A Multicenter Randomized Controlled Trial with 1 to 3 Years of Follow-Up

**DOI:** 10.1155/2017/8017175

**Published:** 2017-09-13

**Authors:** Alessandro Cucchi, Elisabetta Vignudelli, Simonetta Franco, Luca Levrini, Dario Castellani, Luca Pagliani, Massimiliano Rea, Claudio Modena, Giulio Sandri, Carlo Longhi

**Affiliations:** ^1^Department of Biomedical and Neuromotor Sciences (DIBINEM), University of Bologna, 40126 Bologna, Italy; ^2^Department of Surgical and Morphological Science, University of Insubria, 21100 Varese, Italy; ^3^Private Practice, 50129 Firenze, Italy; ^4^Private Practice, 20122 Milano, Italy; ^5^Private Practice, 44121 Ferrara, Italy; ^6^Department of Diagnostic and Surgical Science, University of Genoa, 16126 Genoa, Italy; ^7^Private Practice, 37126 Verona, Italy; ^8^Private Practice, 46035 Mantova, Italy

## Abstract

**Purpose:**

To evaluate the survival, success, and complication rates of tapered double-lead threads single implants, placed in fresh extraction sockets and healed sites of the posterior jaws.

**Methods:**

The enrolled patients were randomly divided into 2 groups: in the test group (TG), all implants were inserted at the time of tooth extraction; in the control group (CG), all implants were placed 3 months after extraction. The implants were followed for a period of 1 to 3 years after loading. The main outcomes were implant survival, complications, and implant-crown success.

**Results:**

Ninety-two patients had 97 installed implants (49 in the TG, 48 in the CG). Only two implants failed, in the TG; the survival rates were therefore 95.9% (47/49) and 100% (48/48) for TG and CG, respectively. In the surviving implants, no complications were reported, for an implant-crown success of 100%.

**Conclusions:**

Although a significant difference was found in the levels of primary stability between TG and CG, single implants placed in fresh extraction sockets and healed sites of the posterior jaws had similar survival and complication rates. Crestal bone levels and peri-implant bone resorption showed similar values. A longer follow-up period is however required, to confirm these positive outcomes.

## 1. Introduction

Single implants are considered a successful and predictable treatment option to replace teeth that cannot be maintained or restored [1], with high survival/success rates and low incidence of complications in the short- [2, 3] and long-term [4, 5].

Generally, timing of implant placement permits distinguishing between immediate implants, (placed immediately after tooth extraction) and delayed implants, inserted in completely healed bone [6]. Hammerle and colleagues have introduced a detailed classification about timing of implant placement: a type 1 implant is a fixture placed into a fresh extraction socket, immediately after tooth extraction; a type 2 implant is a fixture placed from 4 to 8 weeks after tooth extraction, with complete soft tissue coverage of the socket but without bone healing; a type 3 implant is a fixture placed when the soft tissue healing is completed, with a significant degree of bone healing (3 to 6 months from tooth extraction); and type 4 implant is a fixture placed in a fully healed site (at least 6 months after extraction) [7].

In the last years, several authors have demonstrated that immediate implant placement in fresh extraction sockets can be a successful and predictable surgical procedure [9], both in the anterior [10] and in the posterior regions [11] of jaws; this technique reduces the number of surgical sessions and therefore the treatment time and the related costs, increasing patient acceptance and satisfaction [9–11].

However, the immediate implant placement also presents critical aspects [7, 9]. In fact, the stabilization of the implant in the fresh extraction socket may be technically difficult [9, 12]. It is well known in the scientific literature that primary stability is key for the survival and the success of an implant: in the absence of adequate primary stabilization, in fact, a fixture can fail [12–14]. When placing an implant into a fresh extraction socket, the incongruity of size and shape between the fixture and the alveolus can represent a problem [9, 12]. In fact, if in healed sites primary stabilization is obtained by intimate contact between the fixture and the bone, in postextraction sockets residual bony defects always remain around implants [7, 10, 12]. In general, the stabilization of the fixture in the postextraction sockets is obtained by deepening the preparation of the implant site 3-4 mm apically or underpreparing the site, with respect to the implant diameter [15, 16].

Several studies have stressed that primary stability is the key for success in immediate implant placement in the posterior regions, regardless of whether or not there is septal bone, space between the socket walls and the fixture, and grafting material [8, 11, 15]: adequate primary stability was demonstrated to be the most important factor for achievement of osseointegration [12, 13]. Another issue with immediate implant placement is the presence of a gap between the walls of the socket and the body of the fixture [9–11]. To date, in fact, there is no complete agreement among authors about the necessity of filling the gap between implant surface and socket walls after immediate implant placement [17–28]. Some surgical protocols suggest filling the defect if the gap is >2 mm [20, 21] because it may help to foster selective cell colonization and tissue repopulation, to restore the alveolar process [22] especially when socket architecture is critical [23, 24]. In contrast, other surgeons demonstrated successful results leaving the gaps to spontaneous healing without flap elevation, reaching primary closure or grafting [25–27]. They argued that grafting material could increase infection risk because of possible graft exposure and management [8, 12, 28]. Finally, with regard to immediate implant placement into infected sites, the current literature concluded that it can be a viable treatment option if appropriate clinical procedures are followed, such as adequate antibiotic prophylaxis and therapy, meticulous cleaning, and alveolar debridement of the socket, in order to avoid the risk of infection and implant failure [29, 30].

In the last few years, a series of new implant macro- and microtopographies have been introduced on the market: new thread designs have been proposed, with the purpose of better stabilizing the implant in the bone [2, 5, 15, 31], and new implant surfaces have been introduced, with the aim of reducing bone healing times, accelerating, and empowering the osseointegration [32, 33]. This is certainly important to allow the successful insertion of implants in extraction sites, reducing the risk of possible failures during the healing period [31, 32]; however, it is mandatory to obtain appropriate scientific evidence on the clinical performance of these new implant geometries, in order to investigate if they can ensure satisfactory clinical outcomes in different and challenging contexts, including placement in postextraction sockets.

Hence, the aim of the present randomized controlled trial was to evaluate the survival, success, and complication rates of single implants with a new tapered design characterised by double-lead threads, when placed in fresh extraction sockets and healed sites of the posterior jaws.

## 2. Materials and Methods

### 2.1. Patient Enrolment

Over a two-year period (January 2013–December 2015), all patients requiring the extraction of a single tooth in the posterior areas of both jaws (maxillary and mandibular premolars and molars) were considered for possible enrolment in the present multicenter clinical study, involving six different Clinical Centers (two university centers and four dental private practices).

The patient selection was based on the following inclusion criteria: (a) one or more nonrestorable single teeth that had to be extracted and replaced with an implant supported single crown in the posterior maxilla and mandible (only premolar and molar regions); (b) adequate bone volume to place an implant at least 3.7 mm in diameter and 10 mm in length, without bone augmentation procedures; (c) naturally occluding dentition in the opposing jaw; (d) comprehension, acceptance, and full compliance for the treatment and follow-up study protocol.

The exclusion criteria were (a) available bone length < 10 mm and bone width < 4.5 mm; (b) untreated and/or active periodontitis; (c) poor oral hygiene and motivation (full mouth plaque index (FMPI) > 20%; full mouth bleeding index (FMBI) > 20%); (d) heavy smoking habit (>20 cigarettes/day); (e) general contraindication to implant surgery, such as uncontrolled systemic diseases, immunosuppression, and HIV/HCV/HBV infection; (f) chemotherapy and/or irradiation in the head and neck area; (g) treatment with amino-bisphosphonates; (h) pregnancy or nursing; (i) inability to complete the follow-up.

All patients were fully informed about the nature of the present study, the surgical techniques involved, the treatment times, and costs of the therapy; in addition, they were informed about the possible alternative treatment procedures, in accordance with the principles outlined in the Helsinki Declaration (as revised in 2008). Prior to being enrolled in the trial, all patients signed the informed consent form for this study. The study was approved by the Local Ethics Committee at the University of Insubria.

### 2.2. Study Design and Randomization

The present study was designed as a multicenter randomized controlled trial. The randomization method was organized as previously reported [34]. In brief, a researcher (S.F.) who was not directly involved in the treatment of patients worked as a randomization coordinator for the present trial. This researcher prepared a randomization schedule using the freely accessible tools available at http://www.randomization.com/. The sequence generated included randomly varying blocks, of four and eight participants (six and three blocks, respectively). Before tooth extraction, in the moment in which each patient arrived in one of the Clinical Centers involved in the study, each surgeon had to send to the randomization coordinator a text message and an email containing the participant's study ID number. Upon receipt of the text message request, the randomization coordinator consulted the randomization schedule and sent back to the surgeon a text message and an email containing the participant's study ID number and group allocation to the study. According to these indications, all patients were therefore randomly divided into two groups. If the patient was assigned to the* test* group (immediate implant placement), the surgeon had to extract the tooth and to insert the implant in the fresh extraction socket, in the same surgical session. Conversely, if the patient was assigned to the* control *group, the surgeon had to extract the nonrestorable tooth and wait for a period of 4 months, before inserting the implant in the healed ridge, in another surgical session.

### 2.3. Implant System

**Figure 1:**
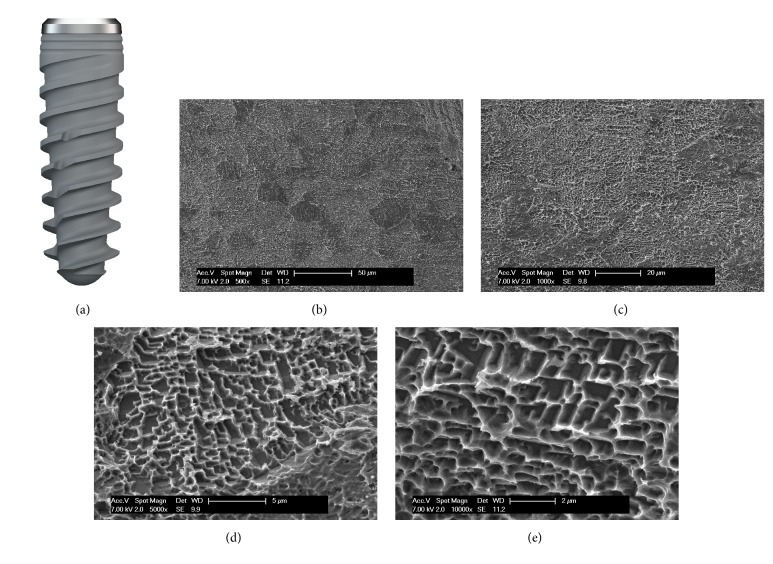
Drawing of the fixture used in this study, with the scanning electron microscopy (SEM) image of the implant surface. The tapered implants used in the present work (BT Safe Bone Level®; Biotec BTK, Dueville, Vicenza, Italy) had double-lead threads. The dual acid etched (DAE) surface of the fixtures had the following roughness parameters: Ra = 1.12 (60.41) *μ*m, Rq = 1.34 (60.69) *μ*m, and Rt = 3.86 (61.40) *μ*m.

The tapered implants used in the present work (BT Safe Bone Level®; Biotec BTK, Dueville, Vicenza, Italy) had double-lead threads with an hexagonal conical connection (11°) and an integrated platform shifting [35]. The dual acid etched (DAE) surface of these implants was the result of treatment with a mixture of strong inorganic acids (H2SO4, H3PO4, HCl, and HF) [36]. The DAE implant surface had the following roughness parameters: Ra (arithmetic mean of the absolute height of all points) = 1.12 (60.41) *μ*m, Rq (square root of the sum of the squared mean difference of all points) = 1.34 (60.69) *μ*m, and Rt (difference between the highest and the lowest points) = 3.86 (61.40) *μ*m [36] (Figure 1). The implants were available in 3.7, 4.1, and 4.8 mm diameter and between 10, 12, and 14 mm in length.

### 2.4. Surgical and Prosthetic Procedure

Patients received professional oral hygiene 1 day before the operation and used chlorhexidine mouthrinse 0.2% for 1 minute, 2 times a day, starting 3 days prior to the intervention and thereafter for 10 days. All patients received prophylactic antibiotic therapy: amoxicillin 2 g 1 hour prior to the intervention and 1 g 6 hours postoperatively.

Following the administration of local anesthesia using articaine with epinephrine 1 : 100.000, a careful circular fiberotomy was performed using periotomes and small elevators to extract the tooth with minimal trauma to the alveolar bone. After all granulation tissue was removed, a periodontal probe was used to verify the dimensions and the integrity of the 4 walls of the fresh sockets. In the* test *group, extraction sockets were prepared for implant placement using standard techniques. Implant sites were prepared with standard drills and the bony walls were followed as a guide. The bone was underdrilled apically to the extraction sockets; at least 3 mm of bone remained beyond the root apex. No countersinking was used. In the* control* group, 3-4 months after tooth extraction was attended for an adequate bone healing, implant placement was performed raising a full-thickness flap extended to the adjacent mesial and distal teeth. Implants were then inserted according to the manufacturer's guidelines. In both* test* and* control* groups, the implants were placed 1 mm below the buccal level of alveolar crest but not more than 2 mm below the lingual/palatal level; when necessary, the surgeon was free to use guided bone regeneration (GBR) with biomaterial, to fill the gap between the implant and the walls of the fresh extraction socket (in the* test* group) or to protect the coronal part of the fixture (in the* control* group). The biomaterial used was a resorbable *β*-tricalcium phosphate [37] (Oxofix®, Biotec BTK, Dueville, Vicenza, Italy, BTK Italy) which could be easily packed in the fresh extraction socket or placed to protect the fixture inserted in a healed site. Sutures were then performed. The patients were instructed to avoid brushing the treated area postoperatively; pain control was provided, as needed, with ibuprofen 600 mg. All implants were left submerged for a period of 3 months; after this undisturbed healing period, all implants were exposed and impressions were taken. A master model was made in the laboratory to produce custom titanium abutments and the definitive metal-ceramic crowns, which were delivered within 1 week (no provisional resin crowns were prepared). All the definitive restorations were single crowns, placed in occlusion. Static and dynamic occlusion were carefully checked using articulating papers. All patients were enrolled in a 6-month follow-up recall program.

### 2.5. Data Collection

All data were measured and recorded according a previously established protocol.

Before extraction, the following parameters were recorded: name of patient; age and gender; smoking habits; tooth site; reason of extraction; presence/absence of chronic periodontal or endodontic infection; width of keratinized tissue.

In* test* group, before implant placement, integrity of the socket walls, integrity of interradicular septum (where applicable), and buccolingual dimension of sockets were measured. After implant placement, these parameters were recorded: implant size; jumping distance (in mm); insertion torque (IT) value (in Ncm) during the implant insertion; implant stability quotient (ISQ) with resonance frequency analysis (RFA) after implant insertion.

In the* control* group, before implant placement, buccal-lingual dimension of alveolar crest and presence of residual bone defects were measured. While after implant placement, implant size, insertion torque (IT) (in Ncm), and implant stability quotient (ISQ) were recorded. During the healing period all surgical and healing complications were evaluated (neurological and vascular injuries, early or late infection, fistula and suppuration). After second-stage surgery, width of keratinized tissue and implant stability quotient (ISQ) were measured clinically. After functional loading health of soft tissue and baseline crestal bone levels were evaluated for each implant. The same parameters were recorded after 1 to 3 years of follow-up.

#### 2.5.1. Width of Keratinized Mucosa (KM)

Keratinized mucosa (KM) was measured with periodontal probe evaluating the distance between margin gingival and mucogingival line, before tooth extraction and after implant restoration.

#### 2.5.2. Alveolar Dimensions and Jumping Distance

The surgeon evaluated after extraction the integrity of the 4 walls of sockets and measured with periodontal probe buccal-lingual and mesiodistal dimensions of alveolar ridge. Then the jumping distance was measured with periodontal probe evaluating the distance between the implant surface and the buccal wall of socket.

#### 2.5.3. Socket Extraction Type

According to the classification of Smith and Tarnow [8] the choice of implant diameter depended on the gap existing between the socket and bone. Particularly in absence of septal bone a wide-diameter implant was recommended, to engage the periphery of the socket. In the other socket categories in presence of septal bone the surgeon decided to proceed with GBR procedure in either case and placed implant in the center of the socket.

#### 2.5.4. Insertion Torque (IT) and Implant Stability Quotient (ISQ)

The insertion torque (IT) and the implant stability quotient (ISQ) were measured as previously reported [38–40]. The IT was measured by means of the implant motor, at the time of implant placement, while the ISQ was measured at different times (at placement, at the time of loading, and 1 and 3 years after loading) by means of a device (Osstell®, Osstell AB, Gothenburg, Sweden) for resonance frequency analysis (RFA) [38–40]. For each fixture, ISQ values (scaled 1–100) were measured. Measurements were taken twice in the buccolingual direction and in the mesiodistal direction; then means were calculated and rounded to the nearest whole number.

#### 2.5.5. Peri-Implant Bone Resorption (PIBR)

Crestal bone levels around osseointegrated implants were assessed on intraoral radiographs collected for each placed implant, at all time points, using a parallel technique. Close attention to proper positioning of the receptor and X-ray tube was used to have radiographs with the same field of view, the same projection and angulation, and the least possible amount of distortion/deformation. Moreover, if evidence of distortion, deformation, or other alterations was present, a new radiograph was taken to achieve an adequate overlapping with previous images. All radiographs were scanned, digitized, converted to 600 dpi resolution.jpeg images, and analyzed through an image analysis software. The values obtained at baseline were compared with the bone levels measured at follow-up to obtain peri-implant bone resorption (PIBR), as previously reported [41, 42].

### 2.6. Primary Outcomes of the Study

#### 2.6.1. Survival of the Implants

Implant survival was considered a primary outcome of this study. All the implants that were regularly in function and under load at the last clinical and radiographic follow-up control (1 or 3 years after placement, resp.) were considered “survivors.” Conversely, all implants that were not osseointegrated after the first healing period were found clinically mobile at second-stage surgery and were therefore removed and considered “failed”; similarly, al implants that suffered for recurrent and intractable acute infection (peri-implantitis) with massive bone loss and clinical mobility and that had to be consequently removed were considered “failed.” Finally, implants were considered “failed” in case of fracture of the fixture body.

#### 2.6.2. Complications

The biologic complications that affected the implant, without causing the failure of the fixture, were listed among the secondary outcomes of the study. These outcomes included (1) persistent pain or dysesthesia or paresthesia in the implant area; (2) peri-implant mucositis, a biologic condition characterised by an inflammation of the soft tissues around the implant, with spontaneous bleeding or bleeding on probing, and possible exudation, associated with probing pocket depth ≥ 4 mm but without any PIBR [43]; (3) peri-implantitis, a biologic condition characterised by inflammation of the soft tissues around the implant, with spontaneous bleeding or bleeding on probing, and possible exudation and/or suppuration, associated with probing pocket depth ≥ 4 mm with evidence of PIBR > 2.5 mm [43].

All possible prosthetic complications that affected the implant-abutment connection, as well as the prosthetic crown, were considered primary outcomes of this study. In detail, these primary outcomes included (1) abutment screw loosening; (2) abutment screw fracture; (3) fracture of the prosthetic abutment; (4) loss of retention of the crown (decementation); (5) ceramic chipping and/or fracture [41].

Taking into account all the aforementioned elements and possible complications, in accordance with the criteria described by Albrektsson et al. [44] and Buser et al. [45], an implant supported restoration was considered successful, in the absence of any biologic and prosthetic complications, and with PIBR < 1.5 mm during the first year of loading and not exceeding 0.2 mm/year during the following years. This condition was defined as “implant-crown success.”

### 2.7. Statistical Analysis

All data analysis was performed according to a preestablished analysis plan using the SPSS software (SPSS version 16®; SPSS Inc., Chicago, IL, USA). A biostatistician with expertise in dentistry analyzed the data, without knowing the group codes. Patients demographics and implants distribution were studied by means of descriptive statistics, as well as implant survival, incidence of complications, and implant-crown success. Absolute and relative (%) frequency distributions were obtained for all qualitative, nonnumerical variables (such as patients' gender, age, according to age intervals, and smoking habit and as implant site, position, reason for tooth extraction, and presence/absence of endodontic lesion). With regard to this, the differences in the distribution of patients and implants between the two groups (postextraction sockets versus healed sites) were investigated using Fisher's exact test. Conversely, means, standard deviations, and ranges were calculated for all quantitative variables, such as patients' age, width of keratinized mucosa (Km), alveolar dimensions and jumping distance, IT, ISQ, and PIBR. Two-tailed *t*-tests for paired and unpaired samples were used to investigate any differences within and between the two groups as far as CBL, IT, ISQ, KM, and PIBR were concerned. To investigate if, within each group, IT and ISQ were correlated, Pearson's *r* coefficient and the corresponding significance were calculated. The 1- and 3-year implant survival and implant-crown success rates were calculated at the patient and at the implant level, and differences, if any, were investigated using Fisher's exact test. The level of significance was set at *p* < 0.05.

## 3. Results

### 3.1. Distribution of Patients

In total 102 patients were originally enrolled in the planned study protocol for a tooth extraction and implant placement. However, 10 patients were excluded from the study, since they did not attend all the scheduled follow-up sessions and therefore they did not have complete clinical and radiographic data. In the* test* group, four patients were excluded for the following reasons: one patient decided not to restore implant with a definitive crown, and three patients were lost during the follow-up period. In the* control *group, six patients dropped out: two patients decided not to place the implant after the healing period, and four patients were lost during the follow-up period. Finally, 92 patients (43 males and 49 females, mean age 51.0 ± 9.5 years, range 20–79 years) were included in the study and used for a complete data collection and statistical analysis. The overall mean follow-up of implants after functional loading was 24.4 ± 9.3 months (range 12–36 months; median 24 months); similar mean follow-up values were noted in the two study groups (24.5 ± 8.9 months in* test* group and 24.3 ± 9.7 months in the* control *group). The distribution of the patients by gender, age, and smoking habit was reported in Table 1. The groups were uniform in the distribution for gender (*p* = 0.676) and smoking habit (0.779), whereas a statistically significant difference was found between* tests *and* controls* in the distribution of patients by age (*p* < 0.001). In fact, in the* test* group most of the patients (64.6%) were aged between 40 and 59 years, whereas in the* control* group a more uniform distribution was present in the three age intervals.

### 3.2. Distribution of Implants

In total, 97 implants were placed in 92 patients (two patients had multiple indications for implant therapy: in fact, one patient received two implants and another one received three implants). Forty-nine implants were placed in fresh postextraction sockets (*test* group), whereas 48 implants were inserted in healed ridges (*control* group). Forty-three implants were inserted in the maxilla, whereas 54 were inserted in the mandible. The distribution of the implants by site, position, reason for tooth extraction, and presence/absence of endodontic lesion was as summarized in Table 2. In the* test* and* control *groups, the distribution of the implants in three of the different categories (site, position, and reason for tooth extraction) was uniform, since no statistically significant differences in the distribution of the fixtures within these groups were noted. Obviously, in the* control* group, no endodontic lesions were present (in fact, after tooth extraction, an undisturbed healing period of 4 months was planned and respected, before placing an implant), and all the endodontic lesions evidenced in the study (14) were in the* test* group: for this reason, a statistically significant difference was found at this level, among* test* and* control *implants (*p* < 0.001).

### 3.3. Width of Keratinized Tissue

In the* control* group, before tooth extraction, the mean width of KM was 2.5 ± 0.9 mm, at the baseline was 2.2 ± 0.9 mm, and at follow-up was 1.9 ± 0.9 mm. In the* test *group, before tooth extraction, the mean width of KM was 2.4 ± 0.9 mm, at the baseline was 1.8 ± 0.7 mm, and at follow-up was 1.6 ± 0.9 mm. Keratinized mucosa (KM) width was not significantly different between the two groups (*p* = 0.62, *p* = 0.07, and *p* = 0.16 before tooth extraction, at baseline, and at follow-up, resp.).

### 3.4. Alveolar Dimensions and Jumping Distance

In the* control* group, the mesiodistal width of the marginal aspect of the sockets was 7.7 ± 0.9 mm, while the buccal-lingual mean width was 8.2 ± 2.2 mm. In the* test* group, the mesial-distal and buccal-lingual widths of the sockets were 7.8 ± 0.8 mm and 8.6 ± 2.0 mm, respectively. Similar values of socket dimensions were observed in the 2 groups (*p* = 0.28 and *p* = 0.52 for mesial-distal and buccal-lingual width, resp.). Moreover, in the* test *group, the jumping distance at the time of implant placement was 2.3 ± 1.5 mm. At reentry surgery, most of implants (90%) showed a complete bone healing, and only few implants (10%) showed a dehiscence less than 1 mm. No implant had a dehiscence more than 1 mm.

### 3.5. Socket Extraction Type

In total, 55 monoradicular teeth and 42 pluriradicular teeth were extracted in the patients. Regarding pluriradicular teeth, 18 were extracted in the* control* group and 24 in the* test *group. According to the classification of Smith and Tarnow [8] for molar extraction sites, 47.3% of the sockets belonged to type A, 32.7% belonged to type B, and the remnants (20.0%) belonged to type C. The distributions of the socket types in the 2 study groups were reported in Table 3. The two distributions were not significantly different (*p* = 0.73).

### 3.6. IT and RFA

The mean IT values measured were 65.5 ± 20.0 in the* control *group and 53.7 ± 23.2 in the* test* group, while the ISQ values displayed were on average 72.8 ± 9.7 and 63.9 ± 12.6, in the* control *and in the* test* groups, respectively. Primary stability was significantly different between the two groups both as far as IT (*p* = 0.008) and ISQ (*p* < 0.001) were concerned. A significant correlation between ISQ and IT was observed in both groups (*test* group: *r* = 0.8473, *p* < 0.001;* control* group: *r* = 0.7951, *p* < 0.001).

### 3.7. Crestal Bone Levels (CBL) and Peri-Implant Bone Resorption (PIBR)

The mean values of CBL at baseline and after follow-up in the* control *group were 0.5 ± 0.4 mm and 0.9 ± 0.4 mm, respectively. In the* test* group, CBL were on average 0.8 ± 0.4 mm at baseline and 1.2 ± 0.6 mm after follow-up At baseline, CBL was significantly different between the two groups (*p* < 0.001). Within each group, CBL at follow-up was significantly different from that at baseline (*p* < 0.001 in both cases). Moreover, CBL at follow-up was slightly different between the two groups (*p* = 0.046).

The PIBR amounted to 0.5 ± 0.4 mm in the* control* group and to 0.4 ± 0.4 mm in the* test* group. These two values were not significantly different (*p* = 0.38) confirming the absence of any difference between the two study groups about bone loss over the time.

### 3.8. Implant Survival

Since two implants failed to be osseointegrated in the* test* group and all implants were osseointegrated in the* control* group, the implant survival rates were 95.9% (47/49) and 100% (48/48), respectively. This difference was not statistically significant (*p* = 0.49). During the follow-up period, no implant was lost, so these favourable outcomes were confirmed. Both two failed implants were 10 × 4.8 mm in size and were placed in the maxilla in molar regions. Moreover, both postextraction sockets were of type C.

### 3.9. Complications and Implant-Crown Success

**Figure 2:**
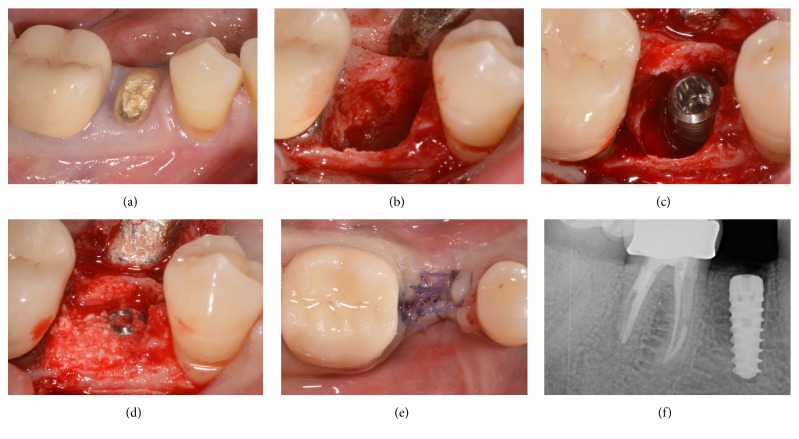
Immediate implant placement of a mandibular second premolar (#45). Preoperative clinical picture of the residual nonrestorable tooth (a); the postextraction socket and the related bone defect (b); implant placement in the postextraction socket (c); bone graft placement in the peri-implant gap (d); primary flap closure and sutures (e); postoperative periapical radiograph after implant surgery (f).

**Figure 3:**
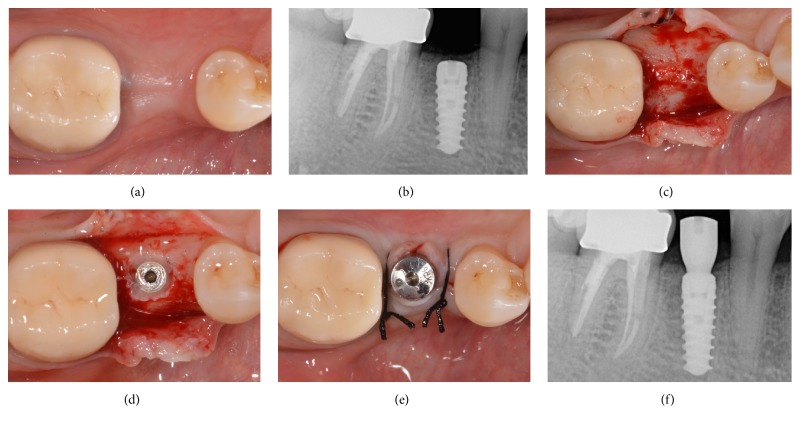
Immediate implant placement of a mandibular second premolar (#45). Soft tissue healing after 3 months (a); periapical radiograph after 3 months of submerged healing (b); second-stage surgery and the alveolar ridge 3 months after implant placement (c); fixture exposure during second-stage surgery (d); placement of the healing abutment and sutures (e); periapical radiograph after insertion of the healing abutment (f).

**Figure 4:**
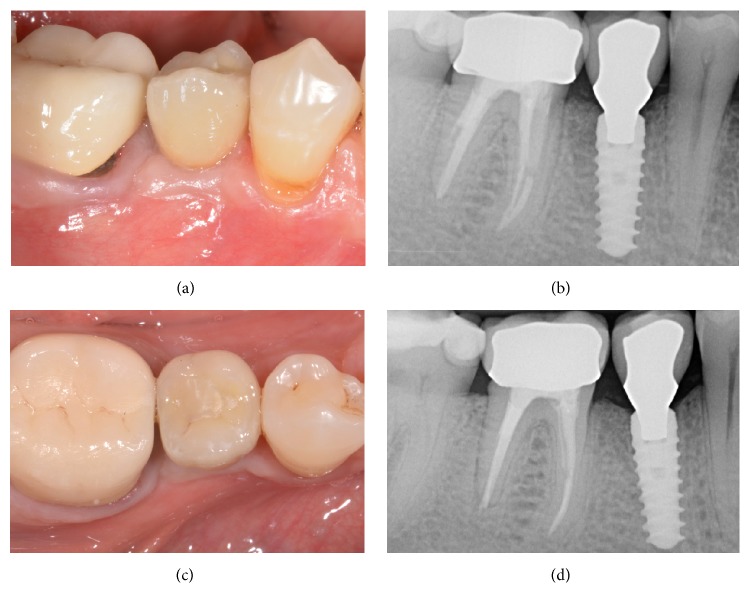
Immediate implant placement of a mandibular second premolar (#45). Final metal-ceramic crown at delivery, lateral view (a); periapical radiograph of the implant at the delivery of the final crown (b); final metal-ceramic crown at delivery, occlusal view (c); periapical radiograph 2 years after implant insertion (d).

**Figure 5:**
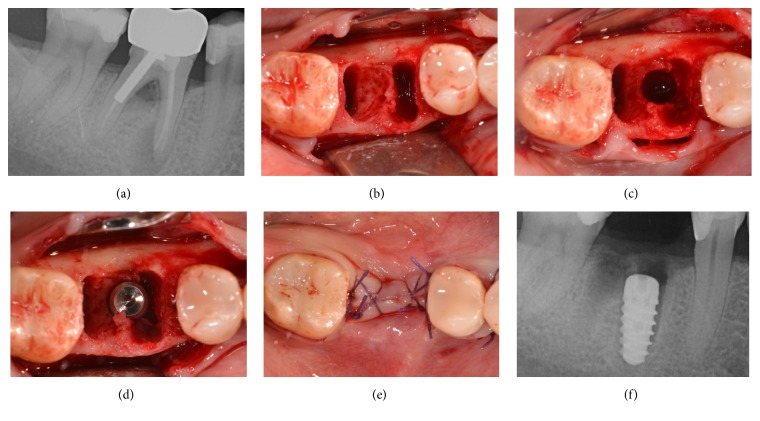
Immediate implant placement of a mandibular first molar (#46). Preoperative periapical radiograph before tooth extraction (a); postextraction socket and bone defect (b); implant site preparation in postextraction socket (c); implant placement in postextraction socket (d); primary flap closure and sutures (e); postoperative radiograph after implant surgery (f).

**Figure 6:**
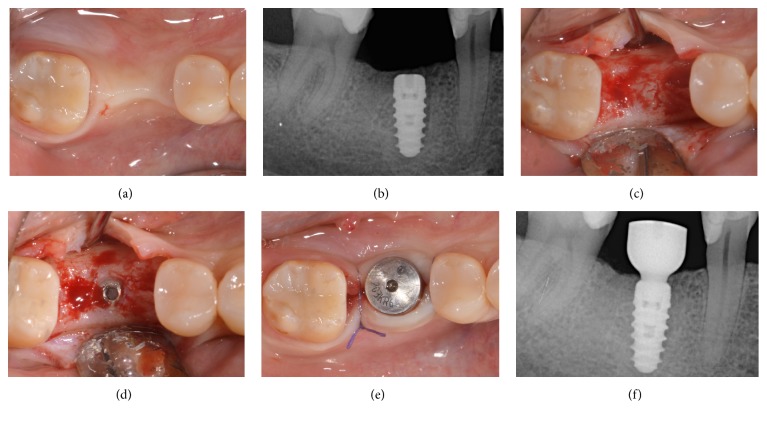
Immediate implant placement of a mandibular first molar (#46). Soft tissue healing 3 months after implant placement (a); periapical radiograph after 3 months of submerged healing (b); second-stage surgery and the alveolar ridge 3 months after implant placement (c); fixture exposure during second-stage surgery (d); placement of the healing abutment and sutures (e); periapical radiograph after insertion of the healing abutment (f).

**Figure 7:**
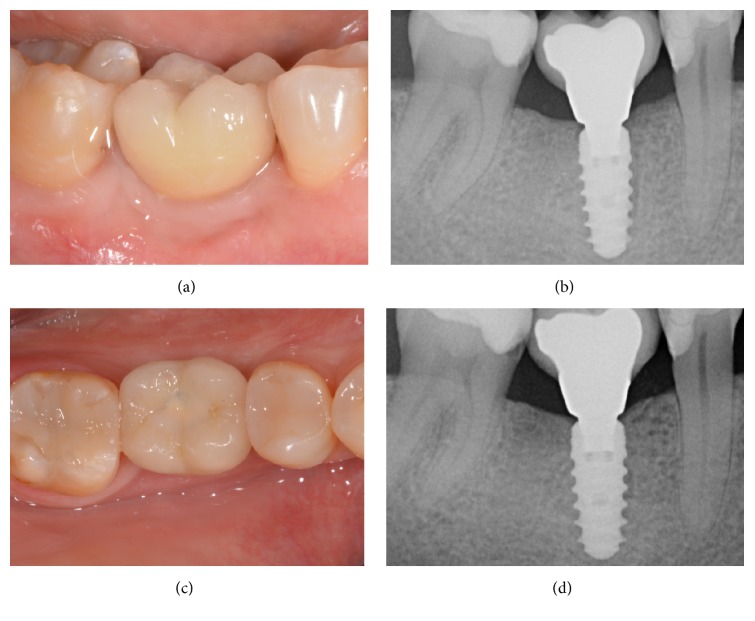
Immediate implant placement of a mandibular first molar (#46). Final metal-ceramic crown at delivery, lateral view (a); periapical radiograph of the implant at the delivery of the final crown (b); final metal-ceramic crown at delivery, occlusal view (c); periapical radiograph 2 years after implant insertion (d).

**Figure 8:**
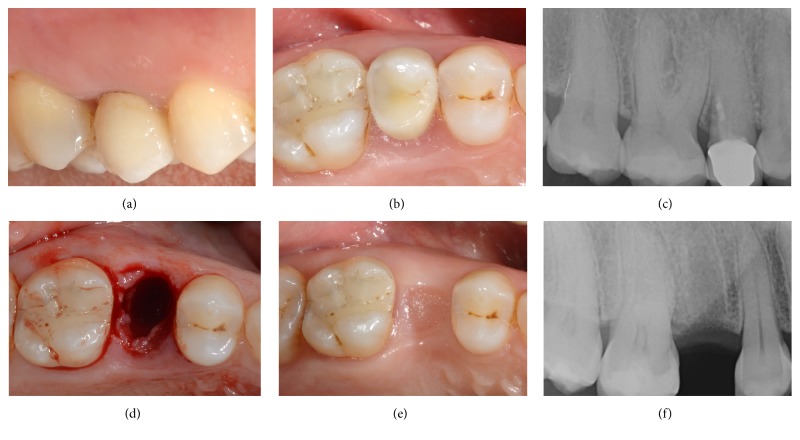
Delayed implant placement of a maxillary second premolar (#15). Preoperative clinical picture, lateral view (a); preoperative clinical picture, occlusal view (b); periapical radiograph before tooth extraction (c); alveolar ridge immediately after extraction (d); alveolar ridge after 3 months of healing (e); periapical radiograph after 3 months of healing (f).

**Figure 9:**
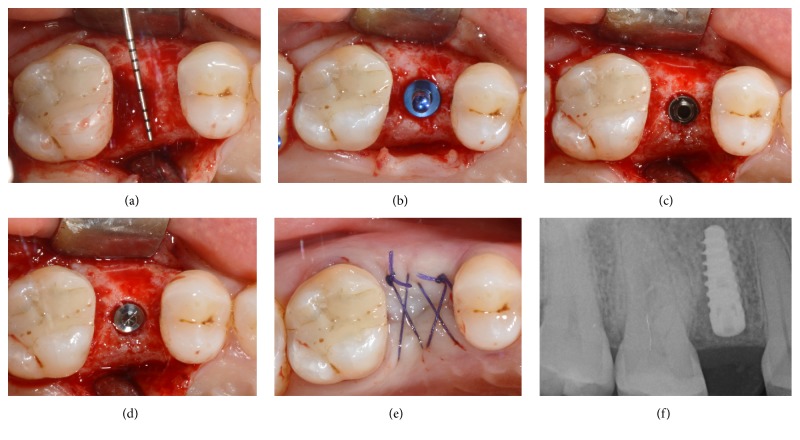
Delayed implant placement of a maxillary second premolar (#15). Alveolar ridge after surgical flap elevation (a); implant site preparation and axis verification (b); implant placement in healed site (c); placement of the cover screw (d); primary flap closure (e); postoperative periapical radiograph (f).

**Figure 10:**
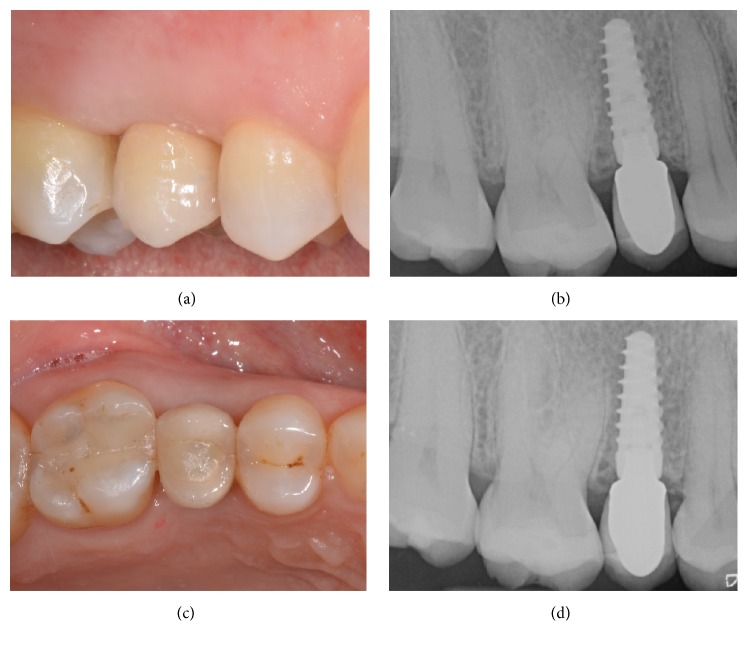
Delayed implant placement of a maxillary second premolar (#15). Final metal-ceramic crown at delivery, lateral view (a); periapical radiograph of the implant at the delivery of the final crown (b); final metal-ceramic crown at delivery, occlusal view (c); periapical radiograph 2 years after implant insertion (d).

No biologic complications were reported during the present study, in both groups. In fact, no postsurgical complications were reported. With regard to soft tissues parameters, at baseline, in the both groups, no sites revealed bleeding on probing (0%). After follow-up period, 17 out of 180 sites evaluated in* control* group had bleeding on probing (9.4%), while in the* test* group, 24 out of 176 sites presented positive bleeding on probing (13.6%). The mean PPD at baseline in the 2 groups were 3.2 ± 1.3 mm and 2.9 ± 1.4 mm, respectively; no significant variations were observed during the follow-up period. No implant showed visible recession of soft tissue over the time, since the marginal level of peri-implant mucosa was located above the margin of implant restoration. Finally, no prosthetic complications were reported during the entire follow-up period, and since no implants showed a PIBR > 1.5 mm during the first year of functional loading and/or exceeded 0.2 mm in each of the following years, an implant-crown success of 100% was found in both groups (Figures 2–10).

## 4. Discussion

Scientific literature showed that implants placed immediately after extraction with or without grafting procedures take advantages of preextraction ridge contours reducing costs, time, second surgery, and discomfort [7, 10–12, 15, 17]. However, still there is not a strong evidence that postextraction implants are absolutely comparable to delayed implants and many issues are still matter of discussion.

In fact, a systematic review published by Esposito et al. in 2010 suggested that immediate implants may be at higher risks of implant failures and complications than delayed implants [46]. However, in that study, the meta-analysis was based on the few trials and for these reasons the authors concluded that there was insufficient evidence to determine the possible advantages or disadvantages of immediate or delayed implants [46].

Conversely, other authors demonstrated that immediate placement of dental implants into postextraction sockets is a successful alternative to the delayed protocol [6, 7, 10–12, 15, 17, 25]. In fact, in these studies, survival rates for immediately placed implants were similar to those for implants placed into healed sites [6, 7, 10–12, 15, 17, 25].

Our present study seems to confirm this evidence. In our study, in fact, favourable survival outcomes were reported for both implants placed in postextraction sockets* (test)* and healed sites* (control)* of the posterior jaws. In fact, only two immediate postextraction implants failed to be osseointegrated: the survival rates were therefore 95.9% (47/49) and 100% (48/48) in the* test* and* control* groups, respectively. The difference in the survival rate within the two groups of implants (*tests* and* controls*) was not significant.

An essential factor for successful immediate or delayed implant placement is the initial stabilization of the implant into the postextraction socket or residual bone [2, 6–8, 11, 13, 15, 17, 31, 39]. Insertion torque (IT) is the easier method to assess the implant stability during implant placement [38]. It is defined as the capacity of the implant to withstand loading in axial, lateral, and rotational directions [38]. It is related to bone quality and quantity bone, implant design and geometry (surface, diameter, length, and type), patient characteristics, and the placement technique used (osteotomy size smaller than the implant diameter, pretapping, or self-tapping) [38–40] and also to the level of primary bone contact also under functional loading and the biomechanical properties of the surrounding bone [38–40]. Immediately implants are given primary stability by the most apical residual alveolar bone while a part of implant surface is surrounded by the clot or the graft and undergoes osseointegration as bone regeneration occurs [6, 11]. For this reason, obtaining primary stability in immediate implants is more difficult than in delayed procedure because there is no presence of a healed residual site for implant anchorage [11].

In our present study, the tapered, double-lead thread design of the implants used here was able to guarantee a satisfactory implant stability, enabling a gradual bone condensation even in low density areas, such as the posterior maxilla. In fact, the mean IT values measured were 65.5 ± 20.0 in the* control *group and 53.7 ± 23.2 in the* test* group; these positive values were further confirmed by RFA method, with mean ISQ values at placement of 72.8 ± 9.7 and 63.9 ± 12.6, in the* control *and in the* test* groups, respectively. A statistically significant difference was found in the levels of primary stability between the test and control implants, for both IT (*p* = 0.008) and ISQ (*p* < 0.001); however, the stability values were satisfactory in both groups. These positives outcomes were probably related to the peculiar design and macrotopography of the implants used in the present study. In particular, the apical deeper cutting threads favoured the insertion and stabilization, while the squared coronal threads seemed to favour bone condensation [31, 35].

However, our study aimed to provide further data that can be useful for better understanding and comparing the clinical performance of implants placed in postextraction and healed sites. Therefore, we have investigated several anatomical, clinical, and radiographic parameters related to these surgical procedures, in order to further validate the immediate approach or to highlight the potential problems related to this. Although many studies confirmed similar survival and success rates of postextraction and delayed implants, there are several factors for evaluation, such as the risk of postoperative infections or complications, the lack of primary stability, the so-called “jumping distance,” and the potential changes of hard and soft tissue dimensions [20, 21, 23]. In fact, it is know that postextraction implants are not able to avoid postextraction alveolar bony changes. A recent systematic review [23] demonstrated that a bone resorption of 0.5–1.0 mm in vertical and horizontal aspects 4–12 months following surgery must be expected. This event could affect osseointegration or cause aesthetic concerns, especially in the maxillary aesthetic zone, which is often characterised by a thin buccal plate [10]. The spontaneous healing of extraction sockets leads to a soft tissue closure after 4–6 weeks, increasing the amount of available keratinized tissue of alveolar ridge. This tissue has a reduction after implant placement, a further reduction after healing screw placement, and a definitive reduction after crown placement due to emergence profile [45, 47–50]. The immediate implant placement can affect the amount of keratinized tissue because of some clinical procedures, such as flap elevation and passivation, primary or secondary closure of sockets, use of grafting biomaterial, or placement of healing screws [51, 52].

The present study reported slight differences about changes of keratinized tissue in the two study groups: in the* control *group, KM had a progressive reduction of about 0.3 mm after implant surgery and about 0.3 mm after prosthetic restoration, while, in the* test *group, KM had a higher reduction (0.6 mm) after implant surgery and a smaller reduction (0.2 mm) after prosthetic rehabilitation. Anyway, the presence of KM more than 1 mm was observed in all implants. Clinical relevance of these findings can be controversial but further studies could give more information about factors influencing the reduction of KM after surgery [12]. No significant gingival recession was noted in both groups after functional loading; but no risk factors for gingival recession were present in the study population. Considering gingival recession, no statistical differences were noted between the two groups. However, several studies demonstrated a significant relationship between recession and thin periodontal biotype, buccal position on implants, and bad oral hygiene [12].

Furthermore, in the present study the classification of Smith and Tarnow [8] was adopted. In this classification, three sockets types (A, B, and C) are presented according to the complete presence (A), partial presence (B), or absence (C) of septal bone. In absence of septal bone (C), as the author suggested [8], a wide-diameter implant was preferred. In the present study, 55 monoradicular teeth and 42 pluriradicular teeth were extracted in the patients; among pluriradicular teeth, 18 were extracted in the* control* group and 24 in the* test *group. According to the aforementioned classification [8] for molar extraction sites, 47.3% of the sockets belonged to type A, 32.7% belonged to type B, and the remnants (20.0%) belonged to type C. No statistically significant differences were noted in the distribution of the implants, according to this classification; however, it is important to underline that all failed implants were placed in maxillary molar regions, in type C extraction sockets: a further study should be required to better investigate the influence of this factor on the osseointegration/survival rates.

Finally, in our study, the PIBR was limited and amounted to 0.5 ± 0.4 mm in the* control* group and to 0.4 ± 0.4 mm in the* test* group. These positive outcomes may be related to the characteristics of the implants used in this study, such as the conical connection (2.6 mm with 11° cone), which has the potential to guarantee an excellent biological seal against the bacterial penetration, eliminating any possible micromovement between the implant and the abutment [35, 41]; also, they may be determined by the integrated platform shifting design of these fixtures that may allow improving soft tissue thickness, giving a barrier against microbial penetration [35, 42]. Moreover, the PIBR values were not significantly different (*p* = 0.38) between* tests* and* controls*, confirming the absence of any difference between the two study groups about bone loss over the time.

As expected and reported by the literature [35, 42], in both groups, most crestal bone loss-level occurred during the course of the first 12 months following baseline. However, few scientific studies underlined that a greater amount of bone loss in immediate implants is more recurring [53, 54]. This finding has been confirmed also in a recent animal study that showed how immediate implant sites had more pronounced bone resorption compared to postextraction sites [54]. Longer follow-up is necessary to assess CBL over time when implants were immediately placed after tooth extraction and to detect any statistical differences.

The limits of the present study are the limited number of patients enrolled, the low number of implants placed, and, most of all, the short follow-up period. A longer follow-up period is mandatory and strictly required, to confirm the positive outcomes emerging from our present study.

## 5. Conclusions

In the present randomized controlled trial on the placement of single implants in postextraction sockets and healed sites of the posterior jaws, 92 patients (43 males, 49 females, mean age 51.0 ± 9.5 years) were installed with 97 implants. Forty-nine implants were placed in fresh postextraction sockets, whereas 48 implants were placed in healed ridges. The implants were followed for a mean period of 24.4 ± 9.3 months (immediate implants: 24.5 ± 8.9 months; delayed implants: 24.3 ± 9.7 months) after loading. At the end of the study, immediate implants achieved similar results to delayed implants, with regard to survival, incidence of complications, and implant-crown success. In fact, only two implants failed in the immediate group: the survival rates were 95.9% (47/49) and 100% (48/48) for immediate and delayed implants, respectively. Finally, in the surviving implants, no complications for reported, for an implant-crown success of 100% after 3 years of loading. However, with immediate implants it was more difficult to achieve primary stability. Studies with longer follow-up are requested to confirm this evidence.

## Figures and Tables

**Table 1:** Patients distribution.

	Test	Control	*p* ^*∗*^	All patients
*Gender*				
Males	21 (43.8%)	22 (50.0%)	0.676	43
Females	27 (56.2%)	22 (50.0%)		49
*Age*				
20–39	12 (25.0%)	10 (22.7%)	<0.001	22
40–59	31 (64.6%)	14 (31.8%)		45
60–79	5 (10.4%)	20 (45.6%)		25
*Smoking habit*				
Yes^*∗∗*^	7 (14.6%)	8 (18.2%)	0.779	15
No	41 (85.4%)	36 (81.8%)		77
All patients	48	44	—	92

*p* ^*∗*^ = Fisher exact test. *∗∗* = patients with ≤20 cigarettes/day were included in the study, conversely patients smoking >20 cigarettes/day were excluded from the study.

**Table 2:** Implants distribution.

	Test	Control	*p* ^*∗*^	All implants
*Site*				
Maxilla	25 (51.0%)	18 (37.5%)	0.221	43
Mandible	24 (49.0%)	30 (62.5%)		54
*Position*				
Premolars	25 (51.0%)	30 (62.5%)	0.307	55
Molars	24 (49.0%)	18 (37.5%)		42
*Reason for tooth extraction*				
Chronic periodontal disease	10 (20.4%)	15 (31.2%)	0.437	25
Destructive caries	23 (46.9%)	21 (43.8%)		44
Root fractures or resorption	16 (32.7%)	12 (25.0%)		28
*Presence of endodontic lesion*				
Yes	14 (28.6%)	0 (0%)	<0.001	14
No	35 (71.4%)	48 (100%)		83
All implants	49	48	—	97

*p* ^*∗*^ = Fisher exact test.

**Table 3:** Sockets distribution in pluriradicular (molar) teeth, according to Smith and Tarnow [8].

Socket type	Test	Control	Total
Type A	12 (21.8%)	14 (25.5%)	26 (47.3%)
Type B	11 (20.0%)	7 (12.7%)	18 (32.7%)
Type C	7 (12.7%)	4 (7.3%)	11 (20.0%)
All sockets	30 (54.5%)	25 (45.5%)	55 (100.0%)

## References

[1] Priest G. (2017). A Current Perspective on Screw-Retained Single-Implant Restorations: A Review of Pertinent Literature. *Journal of Esthetic and Restorative Dentistry*.

[2] Mangano C., Raes F., Lenzi C. (2017). Immediate loading of single implants: a 2-year prospective multicenter study. *The International Journal of Periodontics & Restorative Dentistry*.

[3] Paolantoni G., Marenzi G., Blasi A., Mignogna J., Sammartino G. (2016). Findings of a Four-Year Randomized Controlled Clinical Trial Comparing Two-Piece and One-Piece Zirconia Abutments Supporting Single Prosthetic Restorations in Maxillary Anterior Region. *BioMed Research International*.

[4] Hjalmarsson L., Gheisarifar M., Jemt T. (2016). A systematic review of survival of presented in longitudinal studies with a follow-up of at least 10 years. *European Journal of Oral Implantology*.

[5] Mangano F. G., Shibli J. A., Sammons R. L., Iaculli F., Piattelli A., Mangano C. (2014). Short (8-mm) locking-taper implants supporting single crowns in posterior region: a prospective clinical study with 1-to 10-years of follow-up. *Clinical Oral Implants Research*.

[6] Clementini M., Morlupi A., Agrestini C., Barlattani A. (2013). Immediate versus delayed positioning of dental implants in guided bone regeneration or onlay graft regenerated areas: a systematic review. *International Journal of Oral and Maxillofacial Surgery*.

[7] Chen S. T., Wilson T. G., Hammerle C. H. Immediate early placement of implants following tooth extraction: biologic basis, clinical procedures, and outcomes.

[8] Smith R. B., Tarnow D. P. (2013). Classification of molar extraction sites for immediate dental implant placement: Technical note. *International Journal of Oral and Maxillofacial Implants*.

[9] Clementini M., Tiravia L., De Risi V., Vittorini Orgeas G., Mannocci A., De Sanctis M. (2015). Dimensional changes after immediate implant placement with or without simultaneous regenerative procedures: A systematic review and meta-analysis. *Journal of Clinical Periodontology*.

[10] Mangano F. G., Mastrangelo P., Luongo F., Blay A., Tunchel S., Mangano C. (2017). Aesthetic outcome of immediately restored single implants placed in extraction sockets and healed sites of the anterior maxilla: a retrospective study on 103 patients with 3 years of follow-up. *Clinical Oral Implants Research*.

[11] Tallarico M., Xhanari E., Pisano M., De Riu G., Tullio A., Meloni S. M. (2016). Single post-extractive ultra-wide 7 mm-diameter implants versus implants placed in molar healed sites after socket preservation for molar replacement: 6-month post-loading results from a randomised controlled trial. *European Journal of Oral Implantology*.

[12] Lang N. P., Pun L., Lau K. Y., Li K. Y., Wong M. C. M. (2012). A systematic review on survival and success rates of implants placed immediately into fresh extraction sockets after at least 1 year. *Clinical Oral Implants Research*.

[13] Javed F., Ahmed H. B., Crespi R., Romanos G. E. (2013). Role of primary stability for successful osseointegration of dental implants: factors of influence and evaluation. *Interventional Medicine and Applied Science*.

[14] Renouard F., Nisand D. (2006). Impact of implant length and diameter on survival rates. *Clinical Oral Implants Research*.

[15] Han C. H., Mangano F., Mortellaro C., Park K. B. (2016). Immediate loading of tapered implants placed in postextraction sockets and healed sites. *Journal of Craniofacial Surgery*.

[16] Shadid R. M., Sadaqah N. R., Othman S. A. (2014). Does the implant surgical technique affect the primary and/or secondary stability of dental implants? a systematic review. *International Journal of Dentistry*.

[17] Sanz M., Lindhe J., Alcaraz J., Sanz-Sanchez I., Cecchinato D. (2016). The effect of placing a bone replacement graft in the gap at immediately placed implants: A randomized clinical trial. *Clinical Oral Implants Research*.

[18] Pluemsakunthai W., Le B., Kasugai S. (2015). Effect of buccal gap distance on alveolar ridge alteration after immediate implant placement: A microcomputed tomographic and morphometric analysis in dogs. *Implant Dentistry*.

[19] Maia L. P., Reino D. M., Muglia V. A. (2015). Influence of periodontal tissue thickness on buccal plate remodelling on immediate implants with xenograft. *Journal of Clinical Periodontology*.

[20] Tarnow D. P., Chu S. J. (2011). Human histologic verification of osseointegration of an immediate implant placed into a fresh extraction socket with excessive gap distance without primary flap closure, graft, or membrane: A case report. *International Journal of Periodontics and Restorative Dentistry*.

[21] Khademi A., Shadmehr E., Ajami M., Rismanchian M., Razavi S. M. (2013). Histologic and histomorphometric assessment of implants and periapical tissues when placed in the sockets of extracted teeth, teeth with periapical lesions, and healed lesions: A canine study. *Journal of Oral Implantology*.

[22] Morjaria K. R., Wilson R., Palmer R. M. (2014). Bone healing after tooth extraction with or without an intervention: A systematic review of randomized controlled trials. *Clinical Implant Dentistry and Related Research*.

[23] Lee C.-T., Chiu T.-S., Chuang S.-K., Tarnow D., Stoupel J. (2014). Alterations of the bone dimension following immediate implant placement into extraction socket: systematic review and meta-analysis. *Journal of Clinical Periodontology*.

[24] Paolantonio M., Dolci M., Scarano A. (2001). Immediate implantation in fresh extraction sockets. A controlled clinical and histological study in man. *Journal of Periodontology*.

[25] Mello C., Lemos C., Verri F., dos Santos D., Goiato M., Pellizzer E. (2017). Immediate implant placement into fresh extraction sockets versus delayed implants into healed sockets: A systematic review and meta-analysis. *International Journal of Oral and Maxillofacial Surgery*.

[26] Botticelli D., Berglundh T., Lindhe J. (2004). Hard-tissue alterations following immediate implant placement in extraction sites. *Journal of Clinical Periodontology*.

[27] Covani U., Bortolaia C., Barone A., Sbordone L. (2004). Bucco-lingual crestal bone changes after immediate and delayed implant placement. *Journal of Periodontology*.

[28] Botticelli D., Berglundh T., Buser D., Lindhe J. (2003). The jumping distance revisited: an experimental study in the dog. *Clinical Oral Implants Research*.

[29] Chrcanovic B. R., Martins M. D., Wennerberg A. (2015). Immediate placement of implants into infected sites: a systematic review. *Clinical Implant Dentistry and Related Research*.

[30] de Oliveira-Neto O. B., Barbosa F. T., de Sousa-Rodrigues C. F., de Lima F. J. (2017). Quality assessment of systematic reviews regarding immediate placement of dental implants into infected sites: an overview. *The Journal of Prosthetic Dentistry*.

[31] McCullough J. J., Klokkevold P. R. (2016). The effect of implant macro-thread design on implant stability in the early post-operative period: a randomized, controlled pilot study. *Clinical Oral Implants Research*.

[32] Smeets R., Stadlinger B., Schwarz F. (2016). Impact of dental implant surface modifications on osseointegration. *BioMed Research International*.

[33] Kirmanidou Y., Sidira M., Drosou M.-E. (2016). New Ti-alloys and surface modifications to improve the mechanical properties and the biological response to orthopedic and dental implants: a review. *BioMed Research International*.

[34] Parker M. J., Manan A., Duffett M. (2012). Rapid, easy, and cheap randomization: prospective evaluation in a study cohort. *Trials*.

[35] Dolcini G. A., Colombo M., Mangano C. (2016). From guided surgery to final prosthesis with a fully digital procedure: a prospective clinical study on 15 partially edentulous patients. *International Journal of Dentistry*.

[36] Mangano F. G., Pires J. T., Shibli J. A. (2016). Early bone response to dual acid-etched and machined dental implants placed in the posterior maxilla: a histologic and histomorphometric human study. *Implant Dentistry*.

[37] Giuliani A., Manescu A., Larsson E. (2014). In vivo regenerative properties of coralline-derived (Biocoral) scaffold grafts in human maxillary defects: demonstrative and comparative study with beta-tricalcium phosphate and biphasic calcium phosphate bu synchrotron radiation X-ray microtopograph. *Clinical Implant Dentistry and Related Research*.

[38] Aparicio C., Lang N. P., Rangert B. (2006). Validity and clinical significance of biomechanical testing of implant/bone interface. *Clinical Oral Implants Research*.

[39] Bechara S., Kubilius R., Veronesi G., Pires J. T., Shibli J. A., Mangano F. G. (2016). Short (6-mm) dental implants versus sinus floor elevation and placement of longer (≥10-mm) dental implants: a randomized controlled trial with a 3-year follow-up. *Clinical Oral Implants Research*.

[40] Atieh M. A., Alsabeeha N. H., Payne A. G. (2012). Can resonance frequency analysis predict failure risk of immediately loaded implants?. *International Journal of Prosthodontics*.

[41] Mangano F., Macchi A., Caprioglio A., Sammons R. L., Piattelli A., Mangano C. (2014). Survival and complication rates of fixed restorations supported by locking-taper implants: a prospective study with 1 to 10 years of follow-up. *Journal of Prosthodontics*.

[42] Mangano C., Iaculli F., Piattelli A., Mangano F. (2015). Fixed restorations supported by Morse-taper connection implants: a retrospective clinical study with 10–20 years of follow-up. *Clinical Oral Implants Research*.

[43] Schwarz F., Schmucker A., Becker J. (2015). Efficacy of alternative or adjunctive measures to conventional treatment of peri-implant mucositis and peri-implantitis: a systematic review and meta-analysis. *International Journal of Implant Dentistry*.

[44] Albrektsson T., Zarb G., Worthington P., Eriksson A. R. (1986). The long-term efficacy of currently used dental implants: a review and proposed criteria of success. *The International Journal of Oral & Maxillofacial Implants*.

[45] Buser D., Janner S. F. M., Wittneben J.-G., Brägger U., Ramseier C. A., Salvi G. E. (2012). 10-year survival and success rates of 511 titanium implants with a sandblasted and acid-etched surface: a retrospective study in 303 partially edentulous patients. *Clinical Implant Dentistry and Related Research*.

[46] Esposito M., Grusovin M. G., Polyzos I. P., Felice P., Worthington H. V. (2010). Interventions for replacing missing teeth: dental implants in fresh extraction sockets: immediate, immediate-delayed and delayed implants. *Cochrane Database Systematic Reviews*.

[47] Schropp L., Wenzel A., Kostopoulos L., Karring T. (2003). Bone healing and soft tissue contour changes following single-tooth extraction: a clinical and radiographic 12-month prospective study. *International Journal of Periodontics and Restorative Dentistry*.

[48] Araujo M. G., Sukekava F., Wennström J. L., Lindhe J. (2005). Ridge alterations following implant placement in fresh extraction sockets: an experimental study in the dog. *Journal of Clinical Periodontology*.

[49] Evans C. D. J., Chen S. T. (2008). Esthetic outcomes of immediate implant placements. *Clinical Oral Implants Research*.

[50] Chen S. T., Darby I. B., Adams G. G., Reynolds E. C. (2005). A prospective clinical study of bone augmentation techniques at immediate implants. *Clinical Oral Implants Research*.

[51] Ferrus J., Cecchinato D., Pjetursson E. B., Lang N. P., Sanz M., Lindhe J. (2010). Factors influencing ridge alterations following immediate implant placement into extraction sockets. *Clinical Oral Implants Research*.

[52] Lasella J. M., Greenwell H., Miller R. L. (2003). Ridge preservation with freeze-dried bone allograft and a collagen membrane compared to extraction alone for implant site development: a clinical and histologic study in humans. *Journal of Periodontology*.

[53] Barone A., Aldini N. N., Fini M., Giardino R., Guirado J. L. C., Covani U. (2008). Xenograft versus extraction alone for ridge preservation after tooth removal: a clinical and histomorphometric study. *Journal of Periodontology*.

[54] Vignoletti F., Discepoli N., Müller A., De Sanctis M., Muñoz F., Sanz M. (2012). Bone modelling at fresh extraction sockets: Immediate implant placement versus spontaneous healing. An experimental study in the beagle dog. *Journal of Clinical Periodontology*.

